# The cholesterol lowering efficacy of plant stanol ester yoghurt in a Turkish population: a double-blind, placebo-controlled trial

**DOI:** 10.1186/1476-511X-12-91

**Published:** 2013-06-20

**Authors:** Zehra Buyuktuncer, Mehmet Fisunoğlu, Gulay Sain Guven, Serhat Unal, Halit Tanju Besler

**Affiliations:** 1 Department of Nutrition and Dietetics, Faculty of Health Sciences, Hacettepe University 06100, Sihhiye, Ankara, Turkey; 2 Department of Internal Medicine, Faculty of Medicine, Hacettepe University, Ankara, Turkey

**Keywords:** Plant stanols as esters, Low-fat yoghurt, Hypercholesterolaemia

## Abstract

**Background:**

We evaluated the cholesterol lowering efficacy of low-fat spoonable yoghurt with 1.9 g/d plant stanols as esters on plasma lipid profiles of Turkish subjects with mild to moderate hypercholesterolemia.

**Methods:**

Using a randomised, double-blind, placebo-controlled study design, intervention (n = 35) and control (n = 35) groups consumed either 115 g low-fat yoghurt with 1.9 g/d plant stanols as esters or placebo yoghurt, respectively, for 4 weeks. Seventy subjects with untreated mild to moderate hypercholesterolemia (aged 23-65 years) were recruited. Changes in the lipid profile, including lipoproteins, apolipoproteins, and triglycerides, and anthropometric measurements were monitored at screening, baseline, and at the end of the second, third, and fourth weeks of intervention. The general linear model repeated measures procedure was used to test differences in the repeated continuous variables between study groups.

**Results:**

Serum total cholesterol (4.6%), LDL cholesterol (6.3%), and non-HDL cholesterol (6.2%) concentrations were reduced significantly from baseline in the plant stanol group compared to the control group (p = 0.007, p = 0.005 and p = 0.005, respectively). A variation in the response of serum total and LDL cholesterol between the subjects in plant stanol group was obtained. No clinically significant change in anthropometrical measurements was observed during the intervention.

**Conclusions:**

The spoonable low-fat yoghurt with 1.9 g/d plant stanols as esters lowered total, LDL, and non-HDL cholesterol levels in Turkish subjects with mild to moderate hypercholesterolemia. Nevertheless variation in baseline cholesterol levels, genetic predisposition of the subjects and compliance may contribute to a large individual variability.

## Background

Cardiovascular disease remains the leading cause of mortality in developed countries, but it is also one of the main challenges for developing countries [1,2]. Elevated serum levels of low-density lipoprotein (LDL) cholesterol is a major risk factor for the development of cardiovascular disease. Lifestyle modification, including physical activity and dietary changes, is recognised as the first step towards reducing LDL cholesterol levels in individuals with mild to moderate hypercholesterolaemia. Several dietary factors have been shown to affect the lipid profile, particularly total and LDL cholesterol levels [3]. In addition to the general dietary recommendations for the management of hyperlipidaemia, daily intake of plant sterols or stanols are recommended to lower LDL cholesterol [3,4].

Plant sterols are botanical analogues of cholesterol, and plant stanols are saturated sterols [5]. Due to their structural similarity to cholesterol, plant sterols and stanols were studied initially for their ability to inhibit cholesterol absorption, and their hypocholesterolaemic effects have been known for more than 50 years [6]. Several studies have consistently shown that the intake of 2 g/d of plant sterols/stanols is associated with reduced LDL and total cholesterol levels [7-10]. The National Cholesterol Education Program (NCEP) Adult Treatment Panel (ATP) III report recommends that plant stanols/sterols (2 g/day) should be incorporated into a diet aiming to lower LDL cholesterol by 10-15% [3]. The cholesterol lowering effects of plant stanols/sterols have generally been explained by reduced biliary and dietary absorption of cholesterol from the small intestine. They replace cholesterol in intestinal micelles and less cholesterol is subsequently absorbed; increased plant stanol concentrations within the enterocytes also activate cholesterol efflux back into the intestinal lumen through the ATP binding cassette A1 system [11,12].

Although plant sterols and stanols are naturally found in most plant food sources, the amount in a normal diet is miniscule and unlikely to have a therapeutic effect. With the development of the functional food concept, a new interest in plant stanols was borne when the esterification of these compounds facilitated their inclusion into some food products [13]. Therefore, the daily intake of a recommended amount of plant sterols/stanols can be provided by the consumption of functional foods with plant sterols/stanols [4]. Different food matrixes have been used to add plant sterols/stanols to the diet over the last ten years. However, the number of studies which examined the efficacy of plant stanols in low-fat and aqueous type food carriers (e.g. spoonable yoghurts) is still limited compared to the number of studies conducted using fat-based products (e.g. spreads). Furthermore, the efficacies of plant stanols have been conducted mainly in European or American populations. However, differences in genetic constitution may affect cholesterol metabolism and responses to functional food components with regards to the gene-diet interaction [14,15]. To the best of our knowledge, results from a Turkish population have not yet been reported. Therefore, the present study aimed to evaluate whether low-fat spoonable yoghurt with plant stanols as esters improve the serum lipid profile in Turkish subjects with untreated moderate hypercholesterolemia.

## Results

### Baseline characteristics

The general characteristics of the participants in each study group were similar (Table 1). The mean age in the plant stanol and control groups was 45.5 and 43.5 years, respectively (range 23 to 65 years). No significant differences were found in the frequency of gender or disease, medication use, smoking habits, alcohol consumption or physical activity levels between the study groups. The reported health problems were endocrine diseases (n = 8), hypertension (n = 7), neurological disorders (n = 5), gastrointestinal disorders (n = 4), skeletal muscle disease (n = 4), asthma (n = 4), dermatological diseases (n = 2), psychiatric disorders (n = 2), and renal disease (n = 1). In total, 42.9% of subjects stated that they took medicine regularly; however, none of the medicines were drugs that can affect blood lipid levels. A family history of premature coronary artery disease was present in 48.6% of the patients in the plant stanol group and 57.1% of patients in the control group (*P* = 0.473). No significant differences were found in any of the clinical variables or anthropometric measurements (Table 1). All health screening variables were in reference ranges accepted by Hacettepe University Hospital Laboratory.

**Table 1:** Baseline characteristics and anthropometrical measurements of subjects at screening [%, (mean ± SD) or (median (min-max)]

**Parameter**	**Placebo yoghurt group n = 35**	**Plant stanol yoghurt group n = 35**	**P**
Age (years)	43.5 ± 10.52	45.5 ± 7.06	0.361^b^
Gender (M/F)	14/21	15/20	0.808 ^a^
Smokers (%)	28.6	34.3	0.607 ^a^
Alcohol consumers (%)	40.0	51.4	0.337 ^a^
Physically activity level (PAL)	1.5 ± 0.13	1.5 ± 0.15	0.567 ^b^
**Anthropometrical measurements**			
BMI (kg/m^2^)	28.2 ± 3.19	27.9 ± 3.15	0.685 ^b^
Waist circumference (cm)	93.5 ± 8.93	94.2 ± 10.19	0.775 ^b^
Hip circumference (cm)	103.4 ± 5.44	103.3 ± 8.06	0.924 ^b^
Body Fat Composition (%)	31.3 ± 7.35	31.1 ± 8.11	0.914 ^b^
**Biochemical parameters (mg/dL)**			
Plasma glucose	89.0 (68.0-133.0)	88.0 (64.0-125.00)	0.609 ^c^
Serum alanine amino transferase	20.0 (7.0-59.0)	24.0 (5.0-62.0)	0.948 ^c^
Serum aspartate amino transferase	20.0 (11.0-44.0)	20.0 (10.0-40.0)	0.986 ^c^
Serum glutamyl transferase	19.0 (6.0-61.0)	20.0 (9.0-109.0)	0.204 ^c^
Serum akaline phosphatase	69.0 (37.0-104.0)	71.0 (28.0-153.0)	0.304 ^c^
Serum thyroid-stimulating hormone	1.81(0.54-5.68)	1.96(0.20-5.44)	0.332 ^c^
Serum creatinine	0.87 ± 0.16	0.90 ± 0.15	0.398 ^b^
Serum C-reactive protein	0.32 (0.09-0.89)	0.32 (0.13-2.75)	0.591 ^c^
Total cholesterol	239.0(203.0-288.0)	244.0 (204.0-283.0)	0.488 ^c^
HDL-cholesterol	51.0 (38.0-87.0)	50.0 (31.0-93.0)	0.190 ^c^
LDL-cholesterol	153.1 ± 24.14	159.2 ± 21.9	0.267 ^b^
Triglycerides	141.0 (54.0-370.0)	166.0 (59.0-310.0)	0.226 ^c^
Non-HDL-cholesterol	184.9 ± 21.26	193.17 ± 26.32	0.152 ^b^
Apolipoprotein A1	156.3 ± 20.41	154.8 ± 26.16	0.796 ^b^
Apolipoprotein B	126.0 ± 17.17	131.6 ± 21.69	0.233 ^b^
Lipoprotein(a)	20.8 (5.96-165.0)	20.0 (9.40-153.0)	0.533 ^c^

^a^: Difference between the groups (Pearson Chi-Square test).

^b^: Difference between the groups (Student T-test was used as the data was normally distributed).

^c^: Difference between the groups (Mann-Whitney U test was used as the data was not normally distributed).

### Anthropometric measurements

No clinically significant change was obtained in anthropometric measurements during the intervention period (data not shown).

### Dietary intake

Compliance with the test products was high, with a median value of 96.6% (85.0-100.0) in the control group and of 96.8% (83.9-100.0) in the plant stanol group (*P* = 0.831). Inspection of the study diaries did not reveal any serious deviations from the study protocol. No significant differences were found between the study groups in energy or nutrient intakes during the intervention period. The median daily intake of energy was 2011 kcal (1050.5-3639.7) in the control group, 47% of which was carbohydrates, 14.3% protein, and 36.7% fat; the daily energy intake was 1861.1 kcal (908.9-3097.6) in the plant stanol group, 49.3% of which was carbohydrates, 14.7% protein, and 37.7% fat. The fatty acid intake in the control group was 11.5% for saturated fatty acids, 13.1% for monounsaturated fatty acids, and 10.1% for polyunsaturated fatty acids; the intake was 12%, 13%, and 9% for saturated, monounsaturated, and polyunsaturated fatty acids, respectively, in the plant stanol group. The dietary cholesterol intake was 227 mg/d and 215 mg/d in the control and plant stanol groups, respectively.

### Serum total lipids, lipoproteins, and apolipoproteins

**Figure 1:**
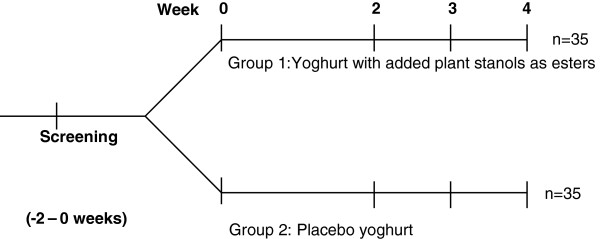
**Individual differences in the LDL cholesterol response (%).** (**a**) plant stanol group and (**b**) placebo yoghurt group.

Lipid profile variables at baseline and the end of the intervention period for both groups are given in Table 2. The baseline was week 0, and the intervention period was the average of the values measured at week 3 and 4. Serum total, LDL, and non-HDL cholesterol concentrations were significantly reduced from the baseline values in the plant stanol group. Table 2 shows a significant reduction in total cholesterol of 4.6%, 6.3% for LDL cholesterol, and 6.2% for non-HDL cholesterol (mean difference between the groups: LDL -6.34%, p = 0.016; total cholesterol -4.54%, p = 0.018; non-HDL -6.20%, p = 0.005) in the plant stanol group compared to the control group. The tendency to decrease serum HDL cholesterol and apolipoprotein B concentrations and to increase VLDL cholesterol, triglycerides, and apolipoprotein A1 concentrations in the plant stanol group was not significantly different in the control group. Because broad variation was observed in the lipid profiles of the participants, the individual differences in LDL cholesterol levels, primary outcome variable, are represented in detail in Figure 1.

**Table 2:** **Effects of placebo and plant stanol yoghurt on lipid profile (mean ± SD)**^**a**^

	**Placebo yoghurt group**			**Plant stanol yoghurt group**			**p**^**b**^
	**Baseline**	**Intervention**	**Change (%)**	**Baseline**	**Intervention**	**Change (%)**	
**Total cholesterol (mg/dl)**	233.4 ± 26.83	235.0 ± 23.75	1.2 ± 8.11	241.1 ± 25.81	232.0 ± 22.12	−3.4 ± 7.60	0.007*
**LDL cholesterol (mg/dl)**	154.5 ± 21.16	154.1 ± 18.58	0.5 ± 10.65	157.9 ± 26.40	147.7 ± 24.41	−5.8 ± 10.90	0.005*
**HDL cholesterol (mg/dl)**	51.2 ± 11.36	50.6 ± 11.62	−0.9 ± 10.76	50.1 ± 14.35	49.6 ± 15.79	−1.2 ± 8.87	0.524
**Triglycerides (mg/dl)**	138.5 ± 66.41	151.6 ± 66.45	19.8 ± 53.75	165.9 ± 99.42	174.0 ± 84.62	11.2 ± 27.05	0.606
**VLDL cholesterol (mg/dl)**	27.7 ± 13.27	30.3 ± 13.26	19.8 ± 53.60	33.1 ± 19.88	34.8 ± 16.92	11.3 ± 27.06	0.596
**Non-HDL cholesterol (mg/dl)**	182.2 ± 24.54	184.4 ± 20.86	2.0 ± 12.98	191.0 ± 28.24	182.5 ± 26.45	−4.2 ± 10.73	0.005*
**Apolipoprotein A-I (mg/dl)**	155.9 ± 20.50	154.3 ± 19.28	−0.8 ± 6.02	152.7 ± 26.12	154.4 ± 33.25	1.4 ± 13.17	0.602
**Apolipoprotein B (mg/dl)**	122.1 ± 19.92	121.1 ± 20.28	−0.4 ± 8.72	128.2 ± 20.09	121.0 ± 19.18	−5.0 ± 11.81	0.076
**Lipoprotein-a (mg/dl)**	27.8 ± 26.86	29.3 ± 27.78	4.1 ± 18.13	35.8 ± 31.18	32.6 ± 29.39	−2.8 ± 25.36	0.094

^a^ :Baseline is Week 0, Intervention is the average of Week 3 and Week 4. Value of Week 2 was used in the calculation of p value, but data was not given at table.

^b^ *p<0.05: Group by time interaction (Repeated measured variance of analysis – general linear model).

## Discussion

The cholesterol lowering effects of foods with plant stanols have been shown in previous studies, with a reduction of 8-17 % in LDL cholesterol [7,8,16]. Although the LDL cholesterol response in the present study was low [6.3%] compared to the reduction rates obtained in some meta-analyses, it is supported by other individual studies with similar product, yoghurt [17-19]. The wide variety seen in the LDL cholesterol responses among different studies can be accounted for by the food matrix supplemented with plant stanols, the form of stanol (i.e. free or ester), the dose of stanol, the ingestion of the stanols with or without a meal, the frequency consumption of products with stanols, the background diet, the baseline LDL cholesterol levels, and the subject’s genotype [20].

The plant stanols were added primarily to fat-based products, such as margarine and spreads, due to their lipophilic nature. After Mensink et al. [21] demonstrated that the ability of plant stanols as esters to block intestinal cholesterol absorption is not necessarily compromised by a low-fat food matrix, other foods, such as milk, yoghurt, juices, and cereals, have been used to add plant stanols. In addition to the fat content of the food matrix, the solid or liquid format of the carrier food has been suggested to affect the efficacy of plant stanols [22]. Because low-fat dairy products are healthy options, low-fat yoghurt was chosen for supplementation with plant stanols in the present study. Research has shown that low fat products (e.g. milk and yoghurt drinks) containing plant stanols have similar efficacy on serum cholesterol as products with higher fat content [23-26]. To the best of our knowledge, the efficacy of spoonable low-fat yoghurts with plant sterol or stanols as esters in reducing LDL cholesterol has been reported in a limited number of studies [17-21,27], which showed a broad range for the reduction in serum total cholesterol (-8.7 to -3.3%) and LDL cholesterol (-13.7 to -2.9%) levels. The reduction in total (4.6%) and LDL cholesterol (6.3%) levels obtained in this study were in accordance with those ranges.

Among the previous studies conducted using spoonable low-fat yoghurts supplemented with plant stanols as esters, the highest reduction in LDL cholesterol (13.7%) was shown by Mensink et al. [21]. These previous results suggested a more pronounced effect compared to the 6.3% reduction in LDL cholesterol in our study. However, a few important points need to be considered. First, the study designs were different. Second, the plant stanol dose was 3 g/d in the study by Mensink et al., which is 1.5-times higher than the dose in the present study. Focussing on the dose response of the cholesterol lowering effect of plant stanols, a continuous dose-dependency appears with doses of up to 2 g, and larger doses of up to 3–4 g slightly enhance the effect [28]. Finally, the consumption frequency of low-fat yoghurt was three times per day in the study by Mensink et al., but it was once a day in the present study. The reduction in LDL cholesterol shown in the present study might have been more pronounced if a multiple daily intake model was chosen. On the other hand, the response to the yoghurt single-shot drink was shown to be enhanced when ingested with a meal [23]. The majority of studies have suggested consuming low-fat products with stanol esters with meals. In this study, lunch was chosen for consuming the test products with plant stanols as esters due to the higher fat content of the workplace lunches, which ensures the initiation of bile secretion flow and micelle formation. In contrast to the study by Mensink et al., the reduction levels obtained in the present study were consistent with the results of some other studies [17-19]. A double-blind, crossover trial by Noakes et al. [17] demonstrated that daily intake of low-fat yoghurt providing 1.8 g of plant stanols reduced LDL cholesterol and total cholesterol by 5% and 3.5%, respectively. In addition, Seppo et al. [18] reported a reduction in LDL cholesterol of 4.9% and in total cholesterol of 3.8% with low-fat yoghurt including 2 g/d plant stanols. Despite some differences in study designs, the main influential factors were matched with the present study.

The baseline LDL cholesterol level was also suggested to be a confounding variable in response to plant stanols [29]. In the study by Seppo et al. [18], the cholesterol lowering effect of plant stanols was suggested to be more prominent when the LDL baseline level was ≥135 mg/dl. The baseline LDL cholesterol in the present study was higher than the proposed cut-off values but similar to previous studies [17,18]. Although the effects of plant stanols appear to be independent of the background diet [7,29], the composition of diets in the present study might be a barrier to achieving the higher reduction rates for LDL cholesterol. Because total (38% of energy) and saturated fat (12% of energy) content of the diet in the present study were higher compared to dietary intakes in some previous studies [21]. The variation in the response of LDL cholesterol is also explained by genetic factors. Previous studies suggested that the apolipoprotein E genotype may have little effect on the response of LDL to plant sterols and stanols; however, the effects of other polymorphisms have not been shown [12,15]. The present study suggests that Turkish people as well as European and American populations will benefit from plant stanol intake, regardless of their genetic background.

In addition to the reduction in total and LDL cholesterol, a small and non-significant reduction in apolipoprotein B levels was observed, which is in agreement with an earlier study [18]. In agreement with the results of previous studies [17,18,21,29], changes in many of the parameters of the lipid profile (e.g., HDL, TG, VLDL, apolipoprotein A, apolipoprotein, and lipoprotein-a) were not significant in the present study, whereas the absolute reductions in LDL and total cholesterol levels were almost identical.

## Conclusions

Low-fat yoghurt with 1.9 g/d plant stanols as esters significantly reduced serum total, LDL, and non-HDL cholesterol levels in a moderately hypercholesterolaemic Turkish population. These results suggest that spoonable yoghurt with added plant stanol esters might be a useful tool to prevent more severe hypercholesterolemia and cardiovascular disease in mildly hypercholesterolaemic individuals.

## Methods

### Study population

Subjects with untreated mild to moderate hypercholesterolemia were recruited from outpatient clinics in the Department of Internal Medicine and Department of Nutrition and Dietetics at Hacettepe University Hospital, Ankara. Subjects were screened on the basis of the following selection criteria: age 20 to 70 years and a fasting serum total cholesterol concentration of 205-290 mg/dl. The exclusion criteria were as follows: lipid lowering medication or other medication that could significantly influence the lipid profile, serum fasting triglyceride concentration of > 4.0 mmol/l, abnormal values for health screening variables, severe obesity (Body Mass Index > 35.0 kg/m^2^), history of cardiovascular disease, other chronic disease (e.g., diabetes, cancer, liver or kidney disorder, celiac disease), severe lactose intolerance or hypersensitivity to milk proteins, pregnancy or lactation, excessive alcohol consumption (15 portions of alcohol/week), supplement use (e.g., fish oil) likely to affect the lipid profile, and the use of plant sterol or stanol supplemented products within 30 days. The study protocol was approved by the Ethics Committee of Hacettepe University and signed written consent was obtained from all participants.

### Study design and diet

**Figure 2:**
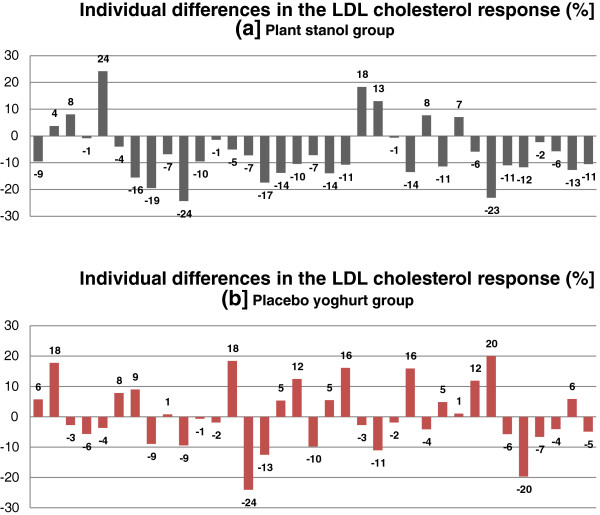
Study design.

The study was a randomised, double-blind, placebo-controlled trial with parallel two-arm design including an intervention and control group (Figure 2). During the 2-week run-in period, all subjects underwent a routine physical examination and biochemical assessment by the research physician, followed by anthropometrical and nutritional assessments by the research dietician. The eligibility of a subject was confirmed by analysis of health screening variables in a fasting blood sample and a structured interview on demographic characteristics, lifestyle behaviours, and medical history. The eligible participants were randomly assigned to the plant stanol group or control yoghurt group using a randomisation list in blocks of four.

During the 4-week intervention period, the plant stanol group consumed 115 g/day low-fat yoghurt with 1.9 g plant stanols as esters, and the control group consumed the same product without plant stanols as a part of lunch every day. The products were produced by Yıldız Holding, packed in blanco packages, labelled, and delivered to the research centre. The energy and nutrient contents of test products are provided in Table 3.

**Table 3:** Energy, nutrient and plant stanol content of test products for 115 g product

	**Plant stanol ester yoghurt**	**Placebo yoghurt**
Energy (kcal)	96.7	84.3
Protein (g)	3.8	3.8
Carbohydrate (g)	17.0	17.0
Fat (g) (excludes plant stanols)	0.1	0.1
Plant stanol ester (g)	3.3	-
Plant stanols (g)	1.9	-

The participants were requested to follow their normal diet and not allowed to consume any additional products with plant stanol or sterols during the study. The use of vitamin, mineral, and/or food supplements was allowed only if the use had been regular before and continued during the study. The background diet and diet during the intervention period were assessed by the research dietician using a 3-day diet diary before week 0, and in week 4. Dietary intake was calculated using the BEBIS dietary analysis computer program (Version 6.0). Dietary intakes, were essentially similar in the two groups at baseline (*P* > 0.05 for all, data not given). The participants were requested to maintain their weight, alcohol consumption, smoking habits, and physical activity during the study. Changes in health and medication during the intervention period were recorded by the participant in the study diary, and the records were checked regularly by the research dietician at each visit. Compliance was assessed by interviewing the patients, reviewing the record of their consumption, and counting the unopened and unconsumed products returned to the clinic. Non-compliance was defined as consuming < 80% of the scheduled serving during the study period.

### Blood sampling and analyses

Blood samples were collected at each visit after a 10-12 h overnight fast, at weeks -2, 0, 2, 3, and 4. Lipid profile and health screening variables, including total cholesterol, LDL- cholesterol, triglycerides, blood count, serum glucose, serum glutamyltransferase, serum creatinine, serum alanine aminotransferase, serum aspartate aminotransferase, serum alkaline phosphatase, serum C-reactive protein, and serum thyroid-stimulating hormone, were determined at the screening visit. Fasting blood samples were collected for analysis of total cholesterol, LDL-cholesterol, high density lipoprotein cholesterol (HDL-cholesterol), very low density lipoprotein cholesterol (VLDL-cholesterol), triglycerides, apolipoprotein A, apolipoprotein B, and lipoprotein-(a) at weeks 0, 2, 3, and 4. Because all samples from each subject, except the screening sample, were analysed in the same analytical run, and the blood samples were stored at –80°C in the Hacettepe University Department of Nutrition and Dietetics Laboratory until analysed after all samples were collected from each individual. Apart from LDL cholesterol, all biomarkers were analysed using routine methods at the Hacettepe University Hospital Biochemistry Laboratory. The estimated LDL cholesterol was calculated using the Friedewald formula [30]. Serum non-HDL cholesterol levels were calculated as the difference between total cholesterol and HDL cholesterol levels. The mean values for the lipid profile variables in weeks 3 and 4 were calculated.

### Anthropometric measurements

Body weight and height were measured using a calibrated digital scale (Seca 220 Scale, Germany). Body mass index (BMI) was calculated by dividing the weight (in kilograms) by the square of the height (in metres). The waist and hip circumferences were measured and the waist-to-hip ratio (WHR) was calculated. Body composition was analysed using the Tanita BC-418 Segmental Body Composition Analyser. All anthropometric measurements were taken by the research dietician.

### Statistical analyses

Statistical analyses were performed using SPSS for Windows (Version 17.0, SPSS Inc, Chicago, USA). Descriptive statistics were computed for discrete variables, and X^2^ statistics were used to examine the differences in these variables between the groups. The normal distribution of continuous variables was evaluated by the Saphiro-Wilk test. The results were presented as means with standard deviations when data were parametric, or medians (minimum-maximum) when data were non-parametric. The general linear model repeated measures procedure was used to test differences in the repeated continuous variables between study groups. *P*-values < 0.05 were regarded to be significant.

## Abbreviations

LDL-cholesterol: Low density lipoprotein cholesterol; VLDL-cholesterol: Very low density lipoprotein cholesterol; HDL-cholesterol: High density lipoprotein cholesterol; TG: Triglycerides; BMI: Body mass index; WHR: Waist-to-hip ratio; NCEP: National cholesterol education program; ATP: Adult treatment panel.

## Competing interests

The authors declare that they have no competing interest.

## Authors’ contributions

Dr ZB was responsible for delivery of the intervention, desk based analyses, interpretation and writing. MF was responsible for delivery of the intervention and data collection. Prof GSG was responsible for eligibility screening, physical examination and clinical evaluations, making trial related medical decisions, documenting in medical notes and medical prescriptions. Prof SU was responsible for confirming eligibility criteria met and making trial related medical decisions. Prof HTB was responsible for overall project supervision, writing and manuscript preparation. All authors critically reviewed the manuscript and approved the final version submitted for publication.

## References

[B1] World Health Organization World Health Report 2003. Shaping the future 2003 Geneva: World Health Organization

[B2] AHA 2006 Diet and lifestyle recommendations. Revision 2006: a scientific statement from the American heart association nutrition committee Circulation 2006 114 82 96 10.1161/CIRCULATIONAHA.106.176158 16785338

[B3] National Cholesterol Education Program (NCEP) Expert Panel on Detection Evaluation, and Treatment of High Blood Cholesterol in Adults (Adult Treatment Panel III) Third report of the national cholesterol education program (NCEP) expert panel on detection, evaluation, and treatment of high blood cholesterol in adults (adult treatment panel III) final report Circulation 2002 106 3143 3421 12485966

[B4] Perk J Backer GD Gohlke H Graham I Reiner Z Verschuren WMM Albus C Benlian P Boysen G Cifkova R Deaton C Ebrahim S Fisher M Germano G Hobbs R Hoes A Karadeniz S Mezzani A Prescott E Ryden L Scherer M Syvanne M Scholte Op Reimer WJM Vrints C Wood D Zamorano JL Zannad F European guidelines on cardiovascular disease prevention in clinical practice (version 2012) Eur Heart J 2012 33 1635 1701 22555213 10.1093/eurheartj/ehs092

[B5] Ellegard LH Andersson SW Normen L Andersson HA Dietary plant sterols and cholesterol metabolism Nutr Rev 2007 65 1 39 45 10.1111/j.1753-4887.2007.tb00266.x 17310858

[B6] Marangoni F Poli A Phytosterols and cardiovascular health Pharmacol Res 2010 61 193 199 10.1016/j.phrs.2010.01.001 20067836

[B7] Katan MB Grundy SM Jones PJ Law ML Miettinen T Paoletti R Efficacy and safety of plant stanols and sterols in the management of blood cholesterol levels Mayo Clin Proc 2003 78 965 978 12911045 10.4065/78.8.965

[B8] Law M Plant sterol and stanol margarines and health BMJ 2000 320 861 864 10.1136/bmj.320.7238.861 10731187 PMC1127206

[B9] AbuMweis SS Vanstone CA Lichtenstein AH Jones PJH Plant sterol consumption frequency affects plasma lipid levels and cholesterol kinetics in humans Eur J Clin Nutr 2009 63 747 755 10.1038/ejcn.2008.36 18523440

[B10] Wu T Fu J Yang Y Zhang L Han J The effects of phytosterols/stanols on blood lipid profiles: a systemic review with meta-analysis Asia Pac J Clin Nutr 2009 18 2 179 186 19713176

[B11] Jones PJ Raeini-Sarjaz M Ntanios FY Vanstone CA Feng JY Parsons WE Modulation of plasma lipid levels and cholesterol kinetics by phytosterol versus phytostanol esters J Lipid Res 2000 41 697 705 10787430

[B12] Plat J Mensink RP Increased intestinal ABC A1 expression contributes to the decrease in cholesterol absorption after plant stanol consumption FASEB J 2002 16 1248 1253 10.1096/fj.01-0718hyp 12153993

[B13] Brufau G Canela MA Rafcas M Phytosterols: physiologic and metabolic aspects related to cholesterol-lowering properties Nutr Res 2008 28 217 225 10.1016/j.nutres.2008.02.003 19083411

[B14] Plat J Mensink RP Relationship of genetic variation in genes encoding apolipoprotein A-IV, scavenger receptor BI, HMG-CoA reductase, CETP and apolipoprotein E with cholesterol metabolism and the response to plant stanol ester consumption Eur J Clin Invest 2002 32 4 242 250 10.1046/j.1365-2362.2002.00982.x 11952809

[B15] Sanchez-Muniz FJ Maki KC Schaefer EJ Ordovas JM Serum lipid and antioxidant responses in hypercholesterolemic men and women receiving plant sterol esters vary by apolipoprotein E genotype J Nutr 2009 139 1 13 19 19056656 10.3945/jn.108.090969

[B16] Musa-Veloso K Poon TH Elliot JA Chung C A comparison of the LDL-cholesterol lowering efficacy of plant stanols and plant sterols over a continuous dose range: Results of a meta-analysis of randomized, placebo-controlled trials Prostaglandins Leukot Essent Fatty Acids 2011 85 1 9 28 10.1016/j.plefa.2011.02.001 21345662

[B17] Noakes M Clifton PM Doornbos AME Trautwein EA Plant sterol esterenriched milk and yoghurt effectively reduce serum cholesterol in modestly hypercholesterolemic subjects Eur J Nutr 2005 44 214 422 10.1007/s00394-004-0513-z 15316827

[B18] Seppo L Jauhiainen T Nevala R Poussa T Korpela R Plant stanol esters in low-fat milk products lower serum total and LDL cholesterol Eur J Nutr 2007 46 2 111 117 10.1007/s00394-006-0639-2 17225918

[B19] Hyun YJ Kim OY Kang JB Lee JH Jang Y Liponkoski L Salo P Plant stanol esters in low-fat yogurt reduces total and low-density lipoprotein cholesterol and low-density lipoprotein oxidation in normocholesterolemic and mildly hypercholesterolemic subjects Nutr Res 2005 25 743 753 10.1016/j.nutres.2005.08.004

[B20] AbuMweis SS Barake R Jones PJ Plant sterols/stanols as cholesterol lowering agents: a meta-analysis of randomized controlled trials Food Nutr Res 2008 52 1 17 10.3402/fnr.v52i0.1811 PMC2596710 19109655

[B21] Mensink RP Ebbing S Lindhout M Plat J van Heugten MMA Effects of plant stanol esters supplied in low fat yoghurt on serum lipids and lipoproteins, non-cholesterol sterols and fat soluble antioxidant concentrations Atherosclerosis 2002 160 205 213 10.1016/S0021-9150(01)00562-7 11755939

[B22] Jones PJ Vanstone CA Raeini-Sarjaz M St-Onge MP Phytosterols in low and non-fat beverages as part of a controlled diet fail to lower plasma lipid levels J Lipid Res 2003 44 1713 1719 10.1194/jlr.M300089-JLR200 12730296

[B23] Doornbos AME Meynen EM Duchateau GSMJE van derKnaap HCM Trautwein EA Intake occasion affects the serum cholesterol lowering of a plant sterol-enriched single-dose yoghurt drink in mildly hypercholesterolaemic subjects Eur J Clin Nutr 2006 60 325 333 10.1038/sj.ejcn.1602318 16234829

[B24] Algorta Pineda J Chinchetru Ranedo MJ Aguirre Anda J Francisco Terreros S Hypocholesteremic effectiveness of a yogurt containing plant stanol esters Rev Clin Esp 2005 205 2 63 66 10.1157/13072497 15766477

[B25] Volpe R Niittynen L Korpela R Sirtori C Bucci A Fraone N Pazzucconi F Effects of yoghurt enriched with plant sterols on serum lipids in patients with moderate hypercholesterolaemia Br J Nutr 2001 86 2 233 239 10.1079/BJN2001395 11502237

[B26] Korpela R Tuomilehto J Hogstrom P Seppo L Piironen V Salo-Vaananen P Toivo J Lamberg-Allardt C Karkkainen M Outila T Sundvall J Vilkkila S Tikkanen MJ Safety aspects and cholesterol-lowering efficacy of low fat dairy products containing plant sterols Eur J Clin Nutr 2006 60 5 633 642 10.1038/sj.ejcn.1602362 16404415

[B27] Clifton PM Noakes M Sullivan D Erichsen N Ross D Annison G Fassoulakis A Cehun M Nestel P Cholesterol-lowering effects of plant sterol esters differ in milk, yoghurt, bread and cereal Eur J Clin Nutr 2004 58 503 509 10.1038/sj.ejcn.1601837 14985690

[B28] Mensink RP de Jong A Lütjohann D Haenen GR Plat J Plant stanols dose-dependently decrease LDL-cholesterol concentrations, but not cholesterol-standardized fat-soluble antioxidant concentrations, at intakes up to 9 g/d Am J Clin Nutr 2010 92 1 24 33 10.3945/ajcn.2009.29143 20504972

[B29] Demonty I Ras RT van der Knaap HC Duchateau GS Meijer L Zock PL Geleijnse JM Trautwein EA Continous dose-response relationship of the LDL-cholesterol-lowering effect of phytosterol intake J Nutr 2009 139 271 284 19091798 10.3945/jn.108.095125

[B30] Friedewald WT Levy RI Fredrickson DS Estimation of theconcentration of low density lipoprotein cholesterol in plasma without use of the preparative ultra centrifugation Clin Chem 1972 18 499 502 4337382

